# Supplementary material for: The Biologically Active Compounds in Fruits of Cultivated Varieties and Wild Species of Apples
Source: Molecules. 2025 Oct 4;30(19):3978. doi: 10.3390/molecules30193978 (PMC12526100; doi:10.3390/molecules30193978)
Supplement: Supplementary file 1 [file molecules-30-03978-s001.zip › Table S1. General table describing the properties of biologically active compounds in apple fruits.pdf]

Table S1. General table describing the properties of biologically active compounds in apple fruits.

Group of compounds	Compound	Properties	Study
Flavonoids and phenylpropanoid derivatives	Cinnamic acid	Antioxidant and anti–inflammatory	[31]
		Antitumor and anticancer	[32, 173]
		Antidiabetic	[33]
		Cardioprotective	[35]
		Hair growth–stimulating	[46]
		Antibacterial	[48]
		Wound healing	[45]
	Caffeic acid	Antioxidant and anti–inflammatory	[40, 174]
		Antitumor and anticancer	[40]
		Cardioprotective	[40, 175]
		Immunostimulating	[40, 175]
		Analgesic	[40, 175]
		Antibacterial	[40, 175]
		Antiviral	[40, 175]
	Chlorogenic acid	Antioxidant and anti–inflammatory	[34, 36, 37, 176]
		Antitumor	[34]
		Antidiabetic	[34, 36, 37]
		Glomerular regeneration	[37]
		Hypoglycaemic	[34, 36]
		Hypocholesterolemic	[34]
		Hypotensive	[34]
		Anti–atherosclerosis	[36]
		Hepatoprotective	[34, 37]
		Antibacterial	[34]
		Antiviral	[34]
	Neochlorogenic acid	Antioxidant and anti–inflammatory	[177, 178]
		Antitumor and anticancer	[178]
		Hepatoprotective	[178]
		Cardioprotective	[95]
	p–Coumaric acid	Antioxidant and anti–inflammatory	[38, 39, 43, 179–181]
		Antitumor and anticancer	[43, 179–181]
		Anti–rheumatic	[43]
		Hepatoprotective	[38, 39, 179, 181]
		Nephroprotective	[38, 39]
	Coumaroylquinic acids	Antioxidant and anti–inflammatory	[182]
		Antitumor and anticancer	[182]
		Cardioprotective	[182]
		Antibacterial	[183]
	Ferulic acid	Antioxidant and anti–inflammatory	[42, 44, 184–188]

		Antitumor and anticancer	[184, 185]
		Hepatoprotective	[184, 186, 187]
		Hypotensive	[184]
		Neuroprotective	[42, 186, 188]
		Antidiabetic	[42, 188]
		Cardioprotective	[42, 184, 187, 188]
		Dermatoprotective	[44, 185]
	Protocatechuic acid	Antioxidant and anti–inflammatory	[189–192]
		Antitumor and anticancer	[189]
		Neuroprotective	[190, 192]
		Cardioprotective	[190, 192]
		Antidiabetic	[190–192]
		Antibacterial	[189, 191]
	Vanillic acid	Antioxidant and anti–inflammatory	[193, 194]
		Neuroprotective	[195]
	Gallic acid	Antioxidant and anti–inflammatory	[196, 197]
		Antitumor/anticancer	[196]
		Hypotensive	[196]
		Hypocholesterolemic	[196]
		Cardioprotective	[196]
	Quercetin	Antioxidant and anti–inflammatory	[198–200]
		Antitumor/anticancer	[200]
		Cardioprotective	[200]
		Hypocholesterolemic	[200]
		Hypotensive	[200]
		Neuroprotective	[198]
		Anti–allergenic / Immunomodulatory	[41]
		Bone–protective (Osteoprotective)	[36]
	Hyperoside	Antioxidant and anti–inflammatory	[201, 202]
		Antitumor and anticancer	[203]
		Cardioprotective	[140]
		Hypocholesterolemic	[204]
		Neuroprotective	[204]
		Hepatoprotective	[201]
		Bone–protective (Osteoprotective)	[140]
	Rutin	Antioxidant and anti–inflammatory	[205]
		Antitumor and anticancer	[36, 205]
		Cardioprotective	[206]
		Hypocholesterolemic	[207]
		Antidiabetic	[207]
		Hypoglycaemic	[207]

		Nephroprotective	[207]
		Neuroprotective	[205]
		Anti–rheumatic	[205]
		Analgesic	[205]
	Kaempferol	Antioxidant and anti–inflammatory	[208–210]
		Antitumor and anticancer	[208, 209]
		Cardioprotective	[211]
		Antidiabetic	[210]
		Hepatoprotective	[212]
		Neuroprotective	[213]
		Antibacterial	[213]
	Catechins	Antioxidant and anti–inflammatory	[36, 214]
		Cardioprotective	[36]
		Anti–allergenic / Immunomodulatory	[36]
		Anti–rheumatic	[36]
		Dermatoprotective	[36]
		Anti–ageing	[36]
	Myricetin	Antioxidant and anti–inflammatory	[215–218]
		Antitumor and anticancer	[219]
		Cardioprotective	[219]
		Antidiabetic	[220–222]
		Hypoglycaemic	[220]
		Hepatoprotective	[223]
		Neuroprotective	[219]
		Bone–protective	[219]
		Antiviral	[224]
	Procyanidins	Antioxidant and anti–inflammatory	[225]
		Antitumor and anticancer	[225]
		Antibacterial	[225, 226]
	Anthocyanins	Antioxidant and anti–inflammatory	[227–232]
		Antitumor and anticancer	[227, 230, 233]
		Cardioprotective	[234, 235]
		Hypotensive	[234, 235]
		Anti–atherosclerosis	[234, 235]
		Antidiabetic	[230, 232]
		Neuroprotective	[230, 236]
	3–Hydroxyphloretin	Antioxidant and anti–inflammatory	[237]
		Antitumor and anticancer	[238, 239]
		Antidiabetic	[240]
		Hypoglycaemic	[240]
		Nephroprotective	[240]
		Dermatoprotective	[240]
		Anti–ageing	[240]

	Phloretin	Antioxidant and anti–inflammatory	[241]
		Antitumor and anticancer	[242]
		Cardioprotective	[241]
		Anti–atherosclerosis	[241]
		Antidiabetic	[241]
		Hypoglycaemic	[241]
		Nephroprotective	[241]
		Neuroprotective	[241]
		Anti–ageing	[241]
		Membrane–modulating / Drug–permeability–enhancing	[36, 49]
	Phloridzin	Antioxidant and anti–inflammatory	[118]
		Antitumor and anticancer	[118]
		Cardioprotective	[118]
		Hypocholesterolemic	[118]
		Hypotensive	[118]
		Anti–atherosclerosis	[118]
		Antidiabetic	[243]
		Hypoglycaemic	[243]
		Nephroprotective	[243]
		Hepatoprotective	[118]
		Immunomodulatory	[118]
		Antibacterial	[118]
		Antiviral	[118]
		Anti–ageing	[118]
		Anti–obesity / Metabolic regulation	[47]
Organic acids	Malic acid	Antioxidant and anti–inflammatory	[52]
		Antitumor and anticancer	[53]
		Neuroprotective	[54]
		Anti–rheumatic	[52]
		Anti–allergenic / Immunomodulatory	[60]
		Dermatoprotective	[61, 245]
		Antiviral	[60]
		Anti–ageing	[61, 245]
		Saliva stimulation / Xerostomia therapy	[246]
		Metabolic support	[52]
	Citric acid	Antioxidant and anti–inflammatory	[58, 247]
		Antitumor and anticancer	[248]
		Cardioprotective	[53, 248]
		Anti–atherosclerosis	[53, 248]
		Nephroprotective	[57]
		Bone–protective	[247]

		Anti–allergenic / Immunomodulatory	[58]
		Anti–ageing	[58, 53]
	Ascorbic acid	Antioxidant and anti–inflammatory	[59, 249]
		Antitumor and anticancer	[59, 250]
		Cardioprotective	[56, 59, 251]
		Hypotensive	[56, 251]
		Anti–atherosclerosis	[56, 251]
		Neuroprotective	[63]
		Bone–protective	[59, 252]
		Anti–allergenic / Immunomodulatory	[59]
		Dermatoprotective	[59, 252]
		Anti–ageing	[59, 63, 249, 252–254]
		Anti–scorbutic	[59]
		Metabolic–protective	[255, 256]
		Embryoprotective	[63]
	Quinic acids	Antioxidant and anti–inflammatory	[257]
		Antitumor and anticancer	[55]
		Cardioprotective	[55]
		Hypocholesterolemic	[55]
		Anti–atherosclerosis	[55, 257]
		Antidiabetic	[55]
		Hypoglycaemic	[55]
		Neuroprotective	[257]
		Anti–allergenic / Immunomodulatory	[257]
		Antibacterial	[55]
		Anti–ageing	[257]
		Anti–obesity	[55]
Pigments	Chlorophylls	Antioxidant and anti–inflammatory	[66, 258]
		Antitumor and anticancer	67
		Cardioprotective	[66, 258]
		Anti–atherosclerosis	[66, 258]
		Anti–allergenic / Immunomodulatory	[66, 258]
	Carotenoids	Antioxidant and anti–inflammatory	[68, 70, 259, 260]
		Antitumor and anticancer	[261–263]
		Cardioprotective	[264]
		Anti–atherosclerosis	[264]
		Antidiabetic	[68]
		Hypoglycaemic	[68]
		Neuroprotective	[69, 71]
		Hepatoprotective	[68]
		Dermatoprotective	[70]
		Anti–ageing	[71, 265]

		Ocular–protective	[71, 265]
Triterpenoids	Ursolic acid and its derivatives 3–Oxo–23–hydroxyurs–12–en–28–oic acid and corosolic acid	Antioxidant and anti–inflammatory	[73, 266]
		Antitumor and anticancer	[74]
		Cardioprotective	[76]
		Hypocholesterolemic	[76]
		Anti–atherosclerosis	[76]
		Hypoglycaemic	[74]
		Neuroprotective	[267]
		Anti–rheumatic	[73]
		Dermatoprotective	[74]
		Anti–ageing	[82]
		Myoprotective / Muscle–enhancing	[82]
	Annurcoic acid	Antioxidant and anti–inflammatory	[268]
	Betulinic acid	Antioxidant and anti–inflammatory	[80]
		Antitumor and anticancer	[269–271]
		Neuroprotective	[270]
		Analgesic	[80]
		Dermatoprotective	[269]
		Antibacterial	[80]
		Antiviral	[80]
		Anthelmintic	[80]
		Antimalarial	[80]
	Euscaphic acid	Antioxidant and anti–inflammatory	[83, 272]
		Antidiabetic	[145]
		Hypoglycaemic	[145]
		Anti–allergenic / Immunomodulatory	[78, 273]
		Dermatoprotective	[78]
		Anti–fatigue	[83]
	Maslinic acid	Antioxidant and an–ti–inflammatory	[77]
		Antitumor and anticancer	[77]
		Cardioprotective	[77]
		Hypocholesterolemic	[77]
		Hypotensive	[77]
		Neuroprotective	[77]
		Antibacterial	[81, 274]
		Anthelmintic	[81, 274]
		Anti–obesity	[81, 274]
	Pomolic acid	Antioxidant and an–ti–inflammatory	[75]
		Antitumor and anticancer	[275–276]
		Cardioprotective	[75]
		Antidiabetic	[75]
		Anti–obesity	[75]

		Bone–protective	[79]
		Antiviral	[277]
		Anti–ageing	[75]
	Pomaceic acid	Insecticidal	[84]
Fatty acids	Linoleic acid	Cardioprotective	[88]
		Neuroprotective	[86]
		Dermatoprotective	[89, 278]
		Hair protection	[89, 278]
		Reproductive health	[89, 278]
	Oleic acid	Antitumor and anticancer	[87, 279]
		Cardioprotective	[87, 280]
		Hypocholesterolemic	[87, 280]
		Hypotensive	[87, 280]
		Antidiabetic	[87, 279]
		Neuroprotective	[87, 279]
		Anti–rheumatic	[87, 279]
		Bone–protective	[87, 279]
		Dermatoprotective	[87, 279]
