# Supplementary material for: The Biologically Active Compounds in Fruits of Cultivated Varieties and Wild Species of Apples
Source: Molecules. 2025 Oct 4;30(19):3978. doi: 10.3390/molecules30193978 (PMC12526100; doi:10.3390/molecules30193978)
Supplement: Supplementary file 1 [file molecules-30-03978-s001.zip › Table S2, S3, S4. Initial general table of biologically active compounds in wild and cultivated apple.pdf]

Table S2. Initial general table of biologically active compounds in cultivated apples

№	Chemical compounds	Cultivated apple varieties					Study
		min, μg/g FW	max, μg/g FW	Mean value, μg/g FW	Total mean value, μg/g FW	Chemical compound content, range (mean value) μg/g FW	
Phenolic compounds							
1	Total phenolic content	404	811	572,3	1016,8	404 – 2170 (1016,8) μg/g FW	[91]
2	Total phenolic content	730	2170	1461,3			[92]
3	Chlorogenic acid	28,25	294,04	108,28	141,06	28,25 – 1104 (141,06) μg/g FW	[96]
4	Chlorogenic acid	121	134	128			[97]
5	Chlorogenic acid		1104	186,9			[98]
6	Caffeic acid	5,9	7,2	6,43	7,47 ‡	1,1 – 18,88 (7,47) μg/g FW ‡	[97]
7	Caffeic acid	1,1	9,2	4,783			[100]
8	Caffeic acid	5,5 ‡	18,88 ‡	11,21			[101]
9	Cinnamic acid	0,02 ‡	1,25 ‡	0,51	0,51 ‡	0,02 – 1,25 (0,51) μg/g FW ‡	[111]
10	Catechins	-	210,9	28,2	36,06	0,45 – 210,9 (36,06) μg/g FW	[98]
11	Catechins	71,1	115,4	90,83			[106]
12	Catechins	4	21	9,5			[107]
13	Catechins	0,45	3,4	15,71			[105]
14	Epicatechin	13,13	97,79	60	88,87	13,13 – 960,4 (88,87) μg/g FW	[96]
15	Epicatechin	-	960,4	120,1			[98]
16	Epicatechin	-	-	86,5			[109]
17	Procyanidins	-	895,3	295,6	185,1	57,8 – 895,3 (185,1) μg/g FW	[98]
18	Procyanidins	57,8	91,4	74,6			[112]
19	Phloretin	0,21 ‡	7,52 ‡	3,28 ‡	19,97 ‡	0,21 – 7,52 (19,97) μg/g	[111]

20	Phloretin	0,41	1,38	0,62 ‡		FW ‡	[131]
21	Phloretin	-	-	56			[132]
22	Phloridzin	5,61	33,15	15,52	25,95	5,61 — 115,5 (25,95) µg/g FW	[96]
23	Phloridzin	-	115,5	19,2			[98]
24	Phloridzin	27,5	58,72	43,14			[115]
25	3-hydroxyphloridzin	-	7,2	1,1	1,1	1,1 µg/g FW	[98]
26	p-Coumaroylquinic acid	0,56	0,74	0,65	9,28	0,56 — 29 (9,28) µg/g FW	[112]
27	p-Coumaroylquinic acid	3,4	7,1	2,06			[105]
28	p-Coumaroylquinic acid	20	29	25,13			[135]
29	Quercetins	23,73	129,32	52,57	40,73	23,73 — 408,4 (40,73) µg/g FW	[96]
30	Quercetins	-	408,4	28,88			[98]
31	Anthocyanins	13	123	53	35,4	4 — 123 (35,4) µg/g FW	[119]
32	Anthocyanins	4	36,7	17,8			[120]
33	Gallic acid	6,3	11,4	8,93	8,93	6,3 — 11,4 (8,93) µg/g FW	[97]
34	Vanillic acid	15,03	15,55	15,29	15,29	15,03 — 15,55 (15,29) µg/g FW	[115]
35	Ferulic acid	1,05	1,37	1,21	1,21	1,05 — 1,37 (1,21) µg/g FW	[115]
36	p-Coumaric acid	8	14,6	10,6	6,41	1,33 — 14,6 (6,41) µg/g FW	[97]
37	p-Coumaric acid	1,33	3,14	2,21			[125]
38	Rutin	38,7	44,4	40,83	41,89	38,7 — 44,4 (41,89) µg/g FW	[97]
39	Rutin	42,33	43,55	42,94			[115]
40	Kaempferol	1,6	2	1,77	28,89	1,6 — 86 (28,89) µg/g FW	[97]
41	Kaempferol	28	86	56			[130]

42	Protocatechuic acid	-	-	4,5	3,43	0,6 — 7,3 (3,43) µg/g FW ‡	[136]
43	Protocatechuic acid	0,6 ‡	7,3 ‡	2,35 ‡			[137]
44	Neochlorogenic acid	0,45	10,37	3,17	1,67	0,45 — 10,37 (1,67) µg/g FW	[139]
45	Neochlorogenic acid	-	-	0,17			[125]
46	Hyperoside	0,6 ‡	1,6 ‡	0,95 ‡	0,95	0,6 — 1,6 (0,95) µg/g FW ‡	[141]
47	Myricetin	5,81	12,1	8,955	8,96	5,81 — 12,1 (8,96) µg/g FW	[115]
Triterpenoids							
48	3-oxo-hydroxy-urs-12-en-28-oic acid_1	-	146,4	21,9	21,9	21,9 µg/g FW	[98]
49	Annurcoic acid	-	437,9	75,9	75,9	75,9 µg/g FW	[98]
50	Betulinic acid	-	31,3	8,1	8,1	8,1 µg/g FW	[98]
51	Corosolic acid	-	97,5	21,2	21,2	21,2 µg/g FW	[98]
52	Euscaphic acid	-	234,3	25,7	25,7	25,7 µg/g FW	[98]
53	Ursolic acid	-	92,7	59,6	59,6	59,6 µg/g FW	[98]
54	Maslinic acid	-	87,4	11,9	11,9	11,9 µg/g FW	[98]
55	Pomolic acid	-	79,7	21,3	21,3	21,3 µg/g FW	[98]
56	Pomaceic acid	-	260,8	5,1	5,1	5,1 µg/g FW	[98]
Fatty acids							
57	Linoleic acid	-	83,4	11,3	11,3	11,3 µg/g FW	[98]
58	Oleic acid	-	80,5	12,4	12,4	12,4 µg/g FW	[98]
Organic acids							
59	Total organic acids	1629	4431,9	2 838,2	5253	1629 — 10100 (5253) µg/g FW	[151]
60	Total organic acids	1720	10100	5253			[152]
61	Malic acid	1542	4353,1	2 736,25	6966	1542 — 17718,1 (6966) µg/g FW	[151]

62	Malic acid	-	17718,1	8682			[98]
63	Malic acid	1720	10100	5250			[152]
64	Citric acid	32,9	551,2	99,43	84,22	32,9 – 551,2 (84,22) μg/g FW	[151]
65	Citric acid	-	-	69			[157]
66	Ascorbic acid	10,48	131,52	44,27	38,39	10,48 – 220,5 (38,39) μg/g FW	[154]
67	Ascorbic acid	-	220,5	32,5			[98]
68	Quinic acids	1,5	571,9	19,17	19,17	1,5 – 571,9 (19,17) μg/g FW	[98]
Pigments							
69	Chlorophylls	-	-	4	3,07 ‡	0,2 – 8,08 (3,07) μg/g FW ‡	[161]
70	Chlorophylls	0,2 ‡	8,08 ‡	2,14 ‡			[160]
71	Carotenoids	-	-	28,2	15,38 ‡	1,328 – 4,95 (15,38) μg/g FW ‡	[161]
72	Carotenoids	1,33 ‡	4,95 ‡	2,56 ‡			[160]

Note: † Average value based on several studies; ‡ Contains data on the chemical composition of apple pulp; φ Human equivalent dose

Table S3. Initial general table of biologically active compounds in wild apples

**№**	**Chemical compounds**	**Wild apple species**					**Study**
		**min, μg/g FW**	**max, μg/g FW**	**average, μg/g FW**	**Total average, μg/g FW**	**Chemical compound content, range (average) μg/g FW**	
Phenolic compounds							
1	Total phenolic content	1460,98	12364,4	4496,405	10465,82	1460,98 – 22753 (10465,8) μg/g FW	[90]
2	Total phenolic content	7309,9	22753	16435,24			[93]
3	Chlorogenic acid	87,17	563,28	264,48	270,93	25,02 – 27459 (270,93) μg/g FW	[96]
4	Chlorogenic acid	25,02	337,84	113,51			[99]
5	Chlorogenic acid	-	27459	434,8			[98]
6	Caffeic acid	-	-	10,6	43,22	0,14 – 138,1 (43,22) μg/g FW	[102]
7	Caffeic acid	99,6	138,1	118,85			[103]

8	Caffeic acid	0,14	0,28	0,21			[99]
9	Cinnamic acid	-	-	0,04	0,04 ‡	0,04 µg/g FW ‡	[111]
10	Catechins	-	131,8	20,9	77,9	20,9 – 134,9 (77,9) µg/g FW	[98]
11	Catechins	-	-	134,9			[102]
12	Epicatechin	62,18	357,17	232,45	2108,98	62,18 – 5816,3 (2108,98) µg/g FW	[96]
13	Epicatechin	-	1567,6	278,2			[98]
14	Epicatechin	-	-	5816,3			[102]
15	Procyanidins	-	2284,4	855,7	345,58 ‡	5,9 – 2284,4 (345,58) µg/g FW ‡	[98]
16	Procyanidins	-	-	114,23 ‡			[111]
17	Procyanidins	5,9	106,3	66,81 ‡			[113]
18	Phloretin	-	-	0,63 ‡	0,63	0,63 µg/g FW ‡	[111]
19	Phloridzin	29,48	82,52	48,08	61,46	29,48 – 430,4 (61,46) µg/g FW	[96]
20	Phloridzin	-	430,4	60,6			[98]
21	Phloridzin	-	-	75,7			[102]
22	3-hydroxyphloridzin	-	54,4	6,4	6,4	6,4 µg/g FW	[98]
23	p-Coumaroylquinic acid	-	-	29 ‡	29 ‡	29 µg/g FW ‡	[135]
24	Quercetins	69,65	205,56	140,85	86,39	69,65 – 290,1 (86,39) µg/g FW	[96]
25	Quercetins	-	290,1	31,92			[98]
26	Anthocyanins	2100	18930	7320	3677,37 ‡	34,74 – 18930 (3677,37) µg/g FW ‡	[90]
27	Anthocyanins	-	-	34,74 ‡			[111]
28	Gallic acid	-	-	547,2	275,11	0,91 – 547,2 (275,2) µg/g FW	[102]
29	Gallic acid	0,91	5,56	3,01			[121]
30	Vanillic acid	-	-	32,34	88,97	11,1 – 314,26 (88,97) µg/g FW	[115]
31	Vanillic acid	11,1	314,26	145,6			[121]
32	Ferulic acid	-	-	14,94	9,46	2,43 – 14,94 (9,46)	[115]

33	Ferulic acid	2,43	5	3,98		µg/g FW	[121]
34	p-Coumaric acid	-	-	37,18	23,76	9,52 — 37,18 (23,76) µg/g FW	[115]
35	p-Coumaric acid	9,52	11,12	10,3375			[121]
36	Rutin	-	-	56,51	134,3	56,51 — 212,09 (134,3) µg/g FW	[115]
37	Rutin	-	-	212,087			[127]
38	Kaempferol	-	-	0,8-20	0,8-20	0,8 — 20 µg/g FW	[129]
39	Protocatechuic acid	1,41	13,36	4,31	3,37	1,41 — 13,36 (3,37) µg/g FW	[99]
40	Protocatechuic acid	1,53	2,98	2,43			[121]
41	Neochlorogenic acid	-	-	5,47	5,47	5,47 µg/g FW	[96]
42	Hyperoside	2,77	23,1	11,99	11,99	2,77 — 23,1 (11,99) µg/g FW	[99]
43	Myricetin	-	-	15,93	15,93	15,93 µg/g FW	[115]
Triterpenoids							
44	3-oxo-hydroxy-urs-12-en-28-oic acid_1	-	56,3	12,8	12,8	12,8 µg/g FW	[98]
45	Annurcoic acid	-	153,8	42,6	42,6	42,6 µg/g FW	[98]
46	Betulinic acid	-	26,4	7,6	7,6	7,6 µg/g FW	[98]
47	Corosolic acid	-	56	16,5	16,5	16,5 µg/g FW	[98]
48	Euscaphic acid	-	171,3	31,6	31,6	31,6 µg/g FW	[98]
49	Ursolic acid	-	114,1	58,1	58,1	58,1 µg/g FW	[98]
50	Maslinic acid	-	49,2	11,6	11,6	11,6 µg/g FW	[98]
51	Pomolic acid	-	36	15,2	15,2	15,2 µg/g FW	[98]
52	Pomaceic acid	-	22,7	2	2	2 µg/g FW	[98]
Fatty acids							
53	Linoleic acid	-	126,4	17,6	17,6	17,6 µg/g FW	[98]
54	Oleic acid	-	181,8	12,5	12,5	12,5 µg/g FW	[98]
Organic acids							
55	Total organic acids	2580	44630	15370	15370	2580 — 44630 (15370) µg/g FW	[152]

56	Malic acid	-	19901,1	11504,3	11550,15	2580 — 29270 (11550,15) μg/g FW	[98]
57	Malic acid	2580	29270	11596			[152]
58	Citric acid	430	24210	3772	2254,5	430 — 24210 (2254,5) μg/g FW	[152]
59	Citric acid	-	-	737			[156]
60	Ascorbic acid	22,07	278,48	78,42	77,41	22,07 — 325 (77,41) μg/g FW	[154]
61	Ascorbic acid	-	325	76,4			[98]
62	Quinic acids	1,3	287,6	18,6	18,6	1,3 — 287,6 (18,6) μg/g FW	[98]
				Pigments			
82	Chlorophylls	-	-	6,51	6,51	6,51 μg/g FW	[162]
84	Carotenoids	-	-	4,8	36,387	0,84 — 99 (36,38) μg/g FW	[162]
85	Carotenoids	0,84	17,58	5,33			[164]
86	Carotenoids	-	-	99			[165]

Note: † Average value based on several studies; ‡ Contains data on the chemical composition of apple pulp; ω Human equivalent dose

The analysis of table data was used to calculate the percentage and X times with which wild and cultivated apple species differ from one another (Supplementary Table S4).

Table S4. Percentage and multiple differences between cultivated and wild apple species in terms of the content of biologically active compounds

№	Chemical compounds	Cultivated apple varieties	Wild apple species	%	X times
1	Phloretin	19,97	0,63	-3070	31,70
2	Cinnamic acid	0,51	0,04	-1175	12,75
3	Pomaceic acid	5,1	2	-155	2,55
4	Annurcoic acid	75,9	42,6	-78	1,78
5	3-oxo-hydroxy-urs-12-en-28-oic acid	21,9	12,8	-71	1,71
6	Pomolic acid	21,3	15,2	-40	1,40
7	Corosolic acid	21,2	16,5	-28	1,28
8	Betulinic acid	8,1	7,6	-7	1,07
9	Quinic acids	19,17	18,6	-3	1,03
10	Maslinic acid	11,9	11,6	-3	1,03
11	Ursolic acid	59,6	58,1	-3	1,03
12	Protocatechuic acid	3,43	3,37	-2	1,02
13	Oleic acid	12,4	12,5	1	1,01
14	Euscaphic acid	25,7	31,6	23	1,23

15	Linoleic acid	11,3	17,6	56	1,56
16	Malic acid	6966	11550,15	66	1,66
17	Myricetin	8,96	15,93	78	1,78
18	Procyanidins	185,1	345,58	87	1,87
19	Chlorogenic acid	141,06	270,93	92	1,92
20	Ascorbic acid	38,39	77,41	102	2,02
21	Chlorophylls	3,07	6,51	112	2,12
22	Quercetins	40,73	86,39	112	2,12
23	Catechins	36,06	77,9	116	2,16
24	Phloridzin	25,95	61,46	137	2,37
25	Carotenoids	15,38	36,38	137	2,37
26	Total organic acids	5253	15370	193	2,93
27	p-Coumaroylquinic acid	9,28	29	213	3,13
28	Rutin	41,89	134,3	221	3,21
29	Neochlorogenic acid	1,67	5,47	228	3,28
30	p-Coumaric acid	6,41	23,76	271	3,71
31	Caffeic acid	7,47	43,22	479	5,79
32	3-hydroxyphloridzin	1,1	6,4	482	5,82
33	Vanillic acid	15,29	88,97	482	5,82
34	Ferulic acid	1,21	9,46	682	7,82
35	Total phenolic content	1016,8	10465,8	929	10,29
36	Hyperoside	0,95	11,99	1162	12,62
37	Epicatechins	88,87	2108,98	2273	23,73
38	Citric acid	84,22	2254,5	2577	26,77
39	Gallic acid	8,93	275,2	2982	30,82
40	Anthocyanins	35,4	3677,37	10288	103,88
