# Supplementary material for: The Biologically Active Compounds in Fruits of Cultivated Varieties and Wild Species of Apples
Source: Molecules. 2025 Oct 4;30(19):3978. doi: 10.3390/molecules30193978 (PMC12526100; doi:10.3390/molecules30193978)
Supplement: Supplementary file 1 [file molecules-30-03978-s001.zip › Table S5, S6, S7 Calculation of effective dose.pdf]

Calculation of effective dose

An effective dose (ED) in pharmacology is the amount of a drug at which a biological reaction occurs [281].

For some substances, information on the effective dose for humans is unavailable, but information on the effective dose for animals is available. Therefore, the human equivalent dose is converted using formulas and tabular data (Eq.3) [282].

$$HED\ (mg/kg) = Animal\ NOAEL\ (mg/kg) \times (Weight\ animal\ [kg]/Weight\ human\ [kg])^{(0.33)}$$

*Note: Animal NOAEL (no observed adverse effect level) - data on the effective dose of the drug in animals without side effec; Weight animal [kg] and Weight human [kg] are taken from the data in Table S5.*

**Table S5.** Reference body weight [282].

| **Species** | **Reference body weight (kg)** |
|---|---|
| Dog | 10 |
| Human | 60 |
| Monkeys | 3 |
| Mouse | 0,020 |
| Rabbit | 1,8 |
| Rat | 0,150 |

Data were collected on the effective dose of the biologically active substances found in apples (Table S6).

**Table S6.** Effective dose of the biologically active compounds found in apples.

| **№** | **Chemical compounds** | **Effective dose** | **Effective dose for humans (taking into account human equivalent dose HED)** | **Study** |
|---|---|---|---|---|
| Phenolic compounds | | | | |
| 1 | Total phenolic content | 220 mg/day | 220 mg per day | [94] |
| 2 | Chlorogenic acid | 13,5 mg per day | 13,5 mg per day | [95] |
| 3 | Caffeic acid | 159,4 mg per day | 159,4 mg per day | [104] |
| 4 | Cinnamic acid | 159,4 mg per day | 159,4 mg per day | [104] |
| 5 | Catechins | 592,9 mg per day | 592,9 mg per day | [108] |
| 6 | Epicatechin | 0,5 mg/kg body weight | 0,5 mg/kg body weight | [110] |
| 7 | Procyanidins (sum) | 704 mg per day | 704 mg per day | [114] |

| | | | | |
|---|---|---|---|---|
| 8 | Phloretin | 10 mg/kg (mouse) | 0,71 mg/kg (HED) | [134] |
| 9 | Phloridzin | 60 mg per day | 60 mg per day | [116] |
| 10 | 3-hydroxyphloridzin | - | - | |
| 11 | p-Coumaroylquinic acid | - | - | |
| 12 | Quercetins | >500 mg/day | >500 mg/day | [117] |
| 13 | Anthocyanins (sum) | 80 mg/day | 80 mg/day | [118] |
| 14 | Gallic acid | 20 mg/kg (mouse) | 1,42 mg/kg (HED) | [122] |
| 15 | Vanillic acid | 50 mg/kg (rat) | 6,92 mg/kg (HED) | [123] |
| 16 | Ferulic acid | 500 - 1000 mg per day | 500 - 1000 mg per day | [124] |
| 17 | p-Coumaric acid | 5 mg/kg (rabbit) | 1,57 mg/kg (HED) | [126] |
| 18 | Rutin | 1 g/day | 1 g/day | [128] |
| 19 | Kaempferol | 5 mg/kg (mouse) | 0,36 mg/kg (HED) | [118] |
| 20 | Protocatechuic acid | 4 mg/kg (rat) | 0,55 mg/kg (HED) | [138] |
| 21 | Neochlorogenic acid | 200 mg per day | 200 mg per day | [95] |
| 22 | Hyperoside | 25 mg/kg (rat) | 3,46 mg/kg (HED) | [140] |
| 23 | Myricetin | 50 mg/kg/day (mouse) | 3,56 mg/kg/day (HED) | [142] |
| Triterpenoids | | | | |
| 24 | 3-oxo-hydroxy-urs-12-en-28-oic acid_1 | - | - | |
| 25 | Annurcoic acid | - | - | |
| 26 | Betulinic acid | 20 mg/kg/day (mouse) | 1,42 mg/kg/day (HED) | [143] |
| 27 | Corosolic acid | 10 mg/kg | 10 mg/kg | [144] |
| 28 | Euscaphic acid | 50 mg/kg (mouse) | 3,56 mg/kg (HED) | [145] |
| 29 | Ursolic acid | 2,65 mg/kg | 2,65 mg/kg | [146] |
| 30 | Maslinic acid | 30 mg/day | 30 mg/day | [147] |
| 31 | Pomolic acid | 0,4 mg/kg (rat) | 0,06 mg/kg (HED) | [148] |
| 32 | Pomaceic acid | - | - | |
| Fatty acids | | | | |

| | | | | |
|---|---|---|---|---|
| 33 | Linoleic acid | 20 g/day | 20 g/day | [149] |
| 34 | Oleic acid | 13,75 - 20,75 g/day | 13,75 - 20,75 g/day | [150] |
| Organic acids | | | | |
| 35 | Total organic acids | - | - | |
| 36 | Malic acid | 1200 mg/day | 1200 mg/day | [153] |
| 37 | Citric acid | 2700 mg/day | 2700 mg/day | [158] |
| 38 | Ascorbic acid | 40 mg/day | 40 mg/day | [155] |
| 39 | Quinic acids | 75 mg/kg (mouse) | 5,34 mg/kg (HED) | [159] |
| Pigments | | | | |
| 40 | Chlorophylls | 150 mg | 150 mg | [163] |
| 41 | Carotenoids | 6,45 mg/day | 6,45 mg/day | [166] |

The effective dose and the content of biologically active substances in cultivated and wild apple fruits were used to calculate the amount of apple fruits required for consumption to achieve this effective dose. Initially, the values of the effective dose of biologically active substances were converted to the general expression μg/day or μg. Then, the value of the effective dose was divided by the content of biologically active substances in the fruits.

**Table S7.** Calculation of the required number of grams of apples for consumption in order to obtain the required amount of biologically active compounds.

| **Chemical compounds** | **Type of apple** | **Chemical compound content, μg/g FW** | **Effective dose for humans** | **Reference human body weight, 60 kg [282]** | **Effective dose for humans, μg/day or μg** | **The required amount of apples for consumption, grams/day or grams** |
|---|---|---|---|---|---|---|
| Phenolic compounds | | | | | | |
| Total phenolic content | Cultivated apple varieties | 1016,8 | 220000 μg/day | | 220000 | 216,37 |
| | Wild apple species | 10465,82 | 220000 μg/day | | 220000 | 21,02 |
| Chlorogenic acid | Cultivated apple varieties | 141,06 | 13500 μg/day | | 13500 | 95,70 |
| | Wild apple species | 270,93 | 13500 μg/day | | 13500 | 49,83 |

| | | | | | | |
|---|---|---|---|---|---|---|
| Caffeic acid | Cultivated apple varieties | 7,47 | 159400 µg/day | | 159400 | 21338,69 |
| | Wild apple species | 43,22 | 159400 µg/day | | 159400 | 3688,11 |
| Catechins | Cultivated apple varieties | 20,2 | 592900 µg/day | | 592900 | 29351,49 |
| | Wild apple species | 77,9 | 592900 µg/day | | 592900 | 7611,04 |
| Epicatechins | Cultivated apple varieties | 88,87 | 500 µg/kg | 60 | 30000 | 337,57 |
| | Wild apple species | 2108,98 | 500 µg/kg | 60 | 30000 | 14,22 |
| Procyanidins | Cultivated apple varieties | 185,1 | 704000 µg/day | | 704000 | 3803,35 |
| | Wild apple species | 345,58 | 704000 µg/day | | 704000 | 2037,15 |
| Phloridzin | Cultivated apple varieties | 25,95 | 60000 µg/day | | 60000 | 2312,14 |
| | Wild apple species | 61,46 | 60000 µg/day | | 60000 | 976,24 |
| Quercetins | Cultivated apple varieties | 40,73 | 500000 µg/day | | 500000 | 12275,96 |
| | Wild apple species | 86,39 | 500000 µg/day | | 500000 | 5787,71 |
| Anthocyanins | Cultivated apple varieties | 35,4 | 80000 µg/day | | 80000 | 2259,89 |
| | Wild apple species | 3677,37 | 80000 µg/day | | 80000 | 21,75 |
| Gallic acid | Cultivated apple varieties | 8,93 | 1420 µg/kg | 60 | 85200 | 9540,87 |
| | Wild apple species | 275,2 | 1420 µg/kg | 60 | 85200 | 309,59 |
| Vanillic acid | Cultivated apple varieties | 15,29 | 6920 µg/kg | 60 | 415200 | 27155,00 |
| | Wild apple species | 88,97 | 6920 µg/kg | 60 | 415200 | 4666,74 |
| Ferulic acid | Cultivated apple varieties | 1,21 | 500000 µg/day | | 500000 | 413223,14 |
| | Wild apple species | 9,46 | 500000 µg/day | | 500000 | 52854,12 |

| | | | | | | |
|---|---|---|---|---|---|---|
| p-Coumaric acid | Cultivated apple varieties | 6,41 | 1570 μg/kg | 60 | 94200 | 14695,79 |
| | Wild apple species | 23,76 | 1570 μg/kg | 60 | 94200 | 3964,65 |
| Rutin | Cultivated apple varieties | 41,89 | 1000000 μg/day | | 1000000 | 23872,05 |
| | Wild apple species | 134,3 | 1000000 μg/day | | 1000000 | 7446,02 |
| Kaempferol | Cultivated apple varieties | 28,89 | 360 μg/kg | 60 | 21600 | 747,66 |
| | Wild apple species | n.d. | 360 μg/kg | 60 | 21600 | |
| Cinnamic acid | Cultivated apple varieties | 0,51 | 159400 μg/day | | 159400 | 312549,02 |
| | Wild apple species | 0,04 | 159400 μg/day | | 159400 | 3985000,00 |
| Phloretin | Cultivated apple varieties | 19,97 | 710 μg/kg | 60 | 42600 | 2133,20 |
| | Wild apple species | 0,63 | 710 μg/kg | 60 | 42600 | 67619,05 |
| Protocatechuic acid | Cultivated apple varieties | 3,43 | 550 μg/kg | 60 | 33000 | 9620,99 |
| | Wild apple species | 3,37 | 550 μg/kg | 60 | 33000 | 9792,28 |
| Neochlorogenic acid | Cultivated apple varieties | 1,67 | 200000 μg/day | | 200000 | 119760,48 |
| | Wild apple species | 5,47 | 200000 μg/day | | 200000 | 36563,07 |
| Hyperoside | Cultivated apple varieties | 0,95 | 3460 μg/kg | 60 | 207600 | 218526,32 |
| | Wild apple species | 11,99 | 3460 μg/kg | 60 | 207600 | 17314,43 |
| Myricetin | Cultivated apple varieties | 8,96 | 3560 μg/kg | 60 | 213600 | 23839,29 |
| | Wild apple species | 15,93 | 3560 μg/kg | 60 | 213600 | 13408,66 |
| Triterpenoids | | | | | | |
| Betulinic acid | Cultivated apple varieties | 8,1 | 1420μg/kg | 60 | 85200 | 10518,52 |
| | Wild apple species | 7,6 | 1420μg/kg | 60 | 85200 | 11210,53 |

| | | | | | | |
|---|---|---|---|---|---|---|
| Corosolic acid | Cultivated apple varieties | 21,2 | 10000 µg/kg | 60 | 600000 | 28301,89 |
| | Wild apple species | 16,5 | 10000 µg/kg | 60 | 600000 | 36363,64 |
| Euscaphic acid | Cultivated apple varieties | 25,7 | 3560 µg/kg | 60 | 213600 | 8311,28 |
| | Wild apple species | 31,6 | 3560 µg/kg | 60 | 213600 | 6759,49 |
| Ursolic acid | Cultivated apple varieties | 59,6 | 2650 µg/kg | 60 | 159000 | 2667,79 |
| | Wild apple species | 58,1 | 2650 µg/kg | 60 | 159000 | 2736,66 |
| Maslinic acid | Cultivated apple varieties | 11,9 | 30000 µg/day | | 30000 | 2521,01 |
| | Wild apple species | 11,6 | 30000 µg/day | | 30000 | 2586,21 |
| Pomolic acid | Cultivated apple varieties | 21,3 | 60 µg/kg | 60 | 3600 | 169,01 |
| | Wild apple species | 15,2 | 60 µg/kg | 60 | 3600 | 236,84 |
| Fatty acids | | | | | | |
| Linoleic acid | Cultivated apple varieties | 11,3 | 20000000 µg/day | | 20000000 | 1769911,50 |
| | Wild apple species | 17,6 | 20000000 µg/day | | 20000000 | 1136363,64 |
| Oleic acid | Cultivated apple varieties | 12,4 | 13750000 µg/day | | 13750000 | 1108870,97 |
| | Wild apple species | 12,5 | 13750000 µg/day | | 13750000 | 1100000,00 |
| Organic acids | | | | | | |
| Malic acid | Cultivated apple varieties | 6966 | 1200000 µg/day | | 1200000 | 172,27 |
| | Wild apple species | 11550,15 | 1200000 µg/day | | 1200000 | 103,89 |
| Ascorbic acid | Cultivated apple varieties | 38,39 | 40000 µg/day | | 40000 | 1041,94 |
| | Wild apple species | 77,41 | 40000 µg/day | | 40000 | 516,73 |
| Citric acid | Cultivated apple varieties | 84,22 | 2700000 µg/day | | 2700000 | 32058,89 |

| | | | | | | |
|---|---|---|---|---|---|---|
| | Wild apple species | 2254,5 | 2700000 µg/day | | 2700000 | 1197,60 |
| Quinic acids | Cultivated apple varieties | 19,17 | 5340 µg/kg | 60 | 320400 | 16713,62 |
| | Wild apple species | 18,6 | 5340 µg/kg | 60 | 320400 | 17225,81 |
| Pigments | | | | | | |
| Chlorophylls | Cultivated apple varieties | 3,07 | 150000 µg/day | | 150000 | 48859,93 |
| | Wild apple species | 6,51 | 150000 µg/day | | 150000 | 23041,47 |
| Carotenoids | Cultivated apple varieties | 15,38 | 6450 µg/day | | 6450 | 419,38 |
| | Wild apple species | 36,38 | 6450 µg/day | | 6450 | 177,30 |
